# The Marine-Derived Oligosaccharide Sulfate (MdOS), a Novel Multiple Tyrosine Kinase Inhibitor, Combats Tumor Angiogenesis both *In Vitro* and *In Vivo*

**DOI:** 10.1371/journal.pone.0003774

**Published:** 2008-11-20

**Authors:** Jingui Ma, Xianliang Xin, Linghua Meng, Linjiang Tong, Liping Lin, Meiyu Geng, Jian Ding

**Affiliations:** Division of Anti-Tumor Pharmacology, State Key Laboratory of Drug Research, Shanghai Institute of Materia Medica, Shanghai Institutes for Biological Sciences, Chinese Academy of Sciences, Shanghai, China; Ordway Research Institute, United States of America

## Abstract

Despite the emerging success of multi-targeted protein tyrosine kinase (PTK) inhibitors in cancer therapy, significant side effects and resistance concerns seems to be avoided unlikely. The aim of the present study was to identify novel multi-targeting PTK inhibitors. The kinase enzymatic activities were measured by enzyme-linked immunosorbent assay (ELISA). The antiproliferative activities in human microvascular endothelial cells (HMECs) were evaluated by sulforhodamine (SRB) assay. The phosphorylation of kinases and their downstream molecules was probed by western blot analysis. The binding mode between MdOS and PTKs was profiled by surface plasmon resonance (SPR) approach and molecular simulation. Tube formation assay, rat aortic ring method and chicken chorioallantoic membrane assay were combined to illustrate the *in vitro* and *in vivo* anti-angiogenic effects. Results indicated that MdOS, a novel marine-derived oligosaccharide sulfate, exhibited a broad-spectrum PTK inhibitory action. At an enzymatic level, MdOS inhibited HER2, EGFR, VEGFR, PDGFR, c-Kit, FGFR1 and c-Src, with little impact on FGFR2. In cellular settings, MdOS inhibited phosphorylation of PTKs, exemplified by HER2, EGFR and VEGFR2, and downstream molecules of Erk1/2 and AKT. Further studies demonstrated that MdOS acted as an ATP-competitive inhibitor via directly binding to the residues of entrance rather than those of the ATP-binding pocket. Furthermore, MdOS inhibited proliferation and tube formation of HMECs, arrested microvessel outgrowth of rat aortic rings and hindered the neovascularization of chick allantoic membrane. Taken together, results presented here indicated that MdOS exhibited anti-angiogenic activity in a PTK-dependent manner and make it a promising agent for further evaluation in PTK-associated cancer therapy.

## Introduction

Protein tyrosine kinases (PTKs) play crucial roles in signal transduction pathways that regulate a number of cellular functions, such as cell proliferation, differentiation, migration and apoptosis[1], [2], [3]. Deregulated expression of PTKs is responsible for tumor development and progression, including hyperproliferation, angiogenesis, invasion and chemotherapy resistance, and have been validated clinically as promising new targets in cancer therapy [2], [4], [5].

Over the past years, there has been a proliferation of agents designed to inhibit single PTK in tumor, including those directed against Bcr-Abl (e.g. imatinib mesylate)[6], epidermal growth factor receptor (EGFR, e.g. erlotinib)[7], HER-2/neu (e.g. trastuzumab)[8]. However, with the exception of a few malignancies that seem to be driven by a single genetic mutation in a gene encoding a signaling protein, most tumors are triggered by multiple mutations in multiple aberrant signaling pathways[9]. Thus, antitumor efficacy of single molecular-targeted agents might be limited. As such, there has been an intriguing interest in discovering and developing novel multi-targeted PTK inhibitors, and most of them focused on small molecular entities. In point of fact, Sunitinib (Sutent, SU11248) and Sorafinib (Nexavar, BAY43-9006), two multi-targeted PTK inhibitors, have shown significant clinical benefits in cancer therapy and approved for the treatment of advanced renal cell carcinoma (RCC) [10], [11], [12], [13].

Since small molecule inhibitors may not only possess potent cytotoxicity and poor solubility, they may also increase the likelihood of development of resistance [14], [15], [16], an exciting challenge of current strategies is to develop new multi-targeting PTK inhibitors with novel scaffolds. To this end, inhibitors are expected to involve those distinct structures from the conventional small molecules.

**Figure 1:**
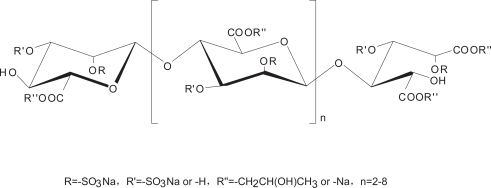
Structure of MdOS.

The oligosaccharides bear unique backbone totally different from that of small molecules that have never been challenged in this setting. Enzyme-linked immunosorbent assay (ELISA), a sensitive and specific assay for the detection and quantification of antigens or antibodies, has been widely used in tyrosine kinase related drug discovery research due to easy-handling and free from radio-contamination, in particular comparing to 32P incorporation [17], [18], [19]. In this paper, with the availability of the newly established in vitro ELISA-based PTK enzymatic profiling assays in our laboratory and in particular, with the marine-derived carbohydrate library in hand, we are encouraged to touch the kind of this class for seeking novel PTK inhibitors. MdOS, a newly semi-synthesized, structurally novel oligosaccharide derived from marine oligomannurarate blocks (Fig. 1), stood out as a potent multi-targeted PTK inhibitor by inhibiting HER2, EGFR, VEGFR2, PDGFR, c-Kit and c-Src. Further studies demonstrate that MdOS exerted anti-angiogenic activities both in vitro and in vivo. All these promise MdOS in particular and, oligosaccharide possible in general, to be a new and hitherto unrecognized scaffold as multi-targeted PTK inhibitors in cancer therapy.

## Results

### MdOS broadly inhibits enzymatic activities of a panel of purified tyrosine protein kinases

The effects of MdOS on the activities of various tyrosine kinases were evaluated using enzyme-linked immunosorbent assays (ELISAs) with purified recombinant proteins. As shown in Table 1, MdOS potently inhibited the kinase activities of HER-2, EGFR and VEGFR2 with IC_50_ values of 0.13, 0.28 and 1.8 µg/ml, respectively. Moreover, MdOS moderately inhibited those of PDGFRβ, c-Kit, c-Src and FGFR1, but had little effect on that of FGFR2. MdOS is, therefore, a multi-targeted tyrosine kinase inhibitor.

**Table 1:** Effects of MdOS on the activity of a panel of tyrosine kinases.

Kinase	IC_50_ (µg/ml)
HER-2	0.13±0.09
EGFR	0.28±0.01
VEGFR2	1.8±0.6
VEGFR1	2.4±0.4
PDGFRβ	1.8±0.3
c-Kit	4±2
c-Src	0.7±0.2
FGFR1	0.68±0.08
FGFR2	>20

Kinases activity was assayed by ELISA. Concentrations that cause 50% inhibition (IC_50_) are shown as mean±SD of three to six separate experiments performed in duplicate. EGFR, epidermal growth factor receptor; VEGFR, vascular endothelial growth factor receptor; PDGFR, platelet derived growth factor receptor; FGFR, fibroblast growth factor receptor.

Given the relative high inhibitory potency of MdOS against HER-2, EGFR and VEGFR2, together with the fact that HER-2, EGFR and VEGFR2 are the most verified targets in cancer therapy, we took these three kinases as representatives to probe MdOS -driven PTKs-associated events.

### MdOS blocks tyrosine kinase phosphorylation and downstream signaling in cells

We next intended to investigate the kinase inhibitory activity of MdOS at cellular level. For this, both naturally and genetically kinase expressing cell lines were selected.

#### MdOS inhibits EGF-induced HER-2 phosphorylation and downstream signaling

**Figure 2:**
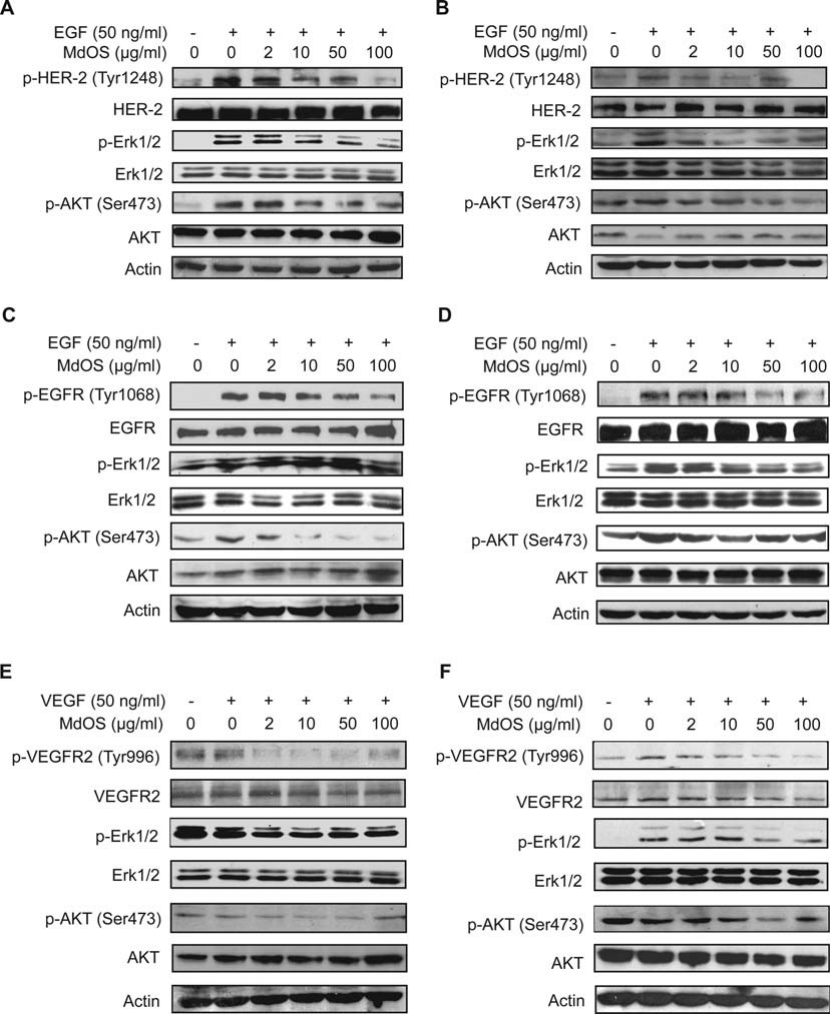
Inhibition of MdOS on cellular kinase phosphorylation and signal transduction. A–B, MdOS blocks HER-2 phosphorylation and its downstream signal transduction in SK-OV-3 cells (A) and NIH-3T3/neu cells (B). C–D, Inhibition of EGF-stimulated EGFR phosphorylation and signal transduction by MdOS in A431 (C) and MDA-MB-468 (D) cells. Serum-starved cells were incubated with indicated concentrations of MdOS for 6 h at 37°C, EGF (50 ng/ml) was added to the cultures during the last 15-min treatment. E–F, Inhibition of VEGF-stimulated VEGFR2 phosphorylation and signal transduction by MdOS. HMEC (E) and NIH-3T3/flk-1 (F) cells were starved, then incubated with indicated concentrations of MdOS for 6 h at 37°C, VEGF (50 ng/ml) was added to the cultures during the last 15-min treatment. Protein samples were separated by SDS-PAGE and probed using the indicated antibodies. Representative data are shown.

The activity of MdOS against HER-2 was followed by measuring receptor autophosphorylation in naturally HER-2-overexpressing SK-OV-3 cells and a newly generated NIH-3T3 cells lines stably expressing HER-2 (NIH-3T3/neu cells). MdOS dose-dependently dephosphorylated HER-2, with a complete abolishment obtained at 100 µg/ml (Fig. 2A). Erk1/2 and AKT, the key downstream molecules of HER-2, playing important roles in cell proliferation and survival, were also significantly dephosphorylated upon MdOS treatment (Fig. 2A). Likewise, MdOS produced a similar pharmacological profile in NIH-3T3/neu cells, with its inhibitory effect observed even at a concentration of 2 µg/ml (Fig. 2B).

#### MdOS inhibits EGF-induced EGFR phosphorylation and downstream signaling

The effects of MdOS on EGFR were measured in naturally EGFR-overexpressing A431 and MDA-MB-468 cells. As shown in Figure 2C, MdOS caused a concentration-dependent abrogation of phosphorylation of EGFR in A431 cells, which was accompanied by a complete abolishment of AKT phosphorylation (10 µg/ml). In contrast, MdOS caused a slight decrease in Erk1/2 phosphorylation (even at 100 µg/ml). In MDA-MB-468 cells, MdOS obviously inactivated EGFR at concentration of 50 µg/ml, and dephosphorylated both Erk1/2 and AKT even at 10 µg/ml (Fig. 2D). All these results indicate that MdOS blocks EGFR and its downstream signaling events.

#### MdOS inhibits VEGF-induced VEGFR2 phosphorylation and signaling

We explored the kinase-inhibitory effects of MdOS on VEGFR2 using HMEC cells that naturally overexpress VEGFR2 and a newly generated NIH-3T3 cells lines stably expressing VEGFR2 (NIH-3T3/flk1 cells). Interestingly, MdOS was noted to be more effective against VEGFR2 in cellular assay compared with the inhibitory potency against HER-2 and EGFR. As shown in Figure 2E, MdOS (2 µg/ml) was able to produce a complete blockage of VEGFR2 phosphorylation, followed by an obvious inhibition on Erk1/2 phosphorylation, but no effect on AKT. MdOS (50 µg/ml) also caused a dramatic abrogation of phosphorylation of VEGFR2 in NIH-3T3/flk-1 cells, accompanied by a complete abolishment of Erk1/2 phosphorylation and an obvious inhibition on AKT phosphorylation (Fig. 2F).

Taken together, MdOS can inhibit phosphorylation of HER-2, EGFR and VEGFR2 intracellularly, with potency against VEGFR2 relatively higher than the other two.

### MdOS targets intracellular tyrosine kinase rather than extracellular growth factors

**Figure 3:**
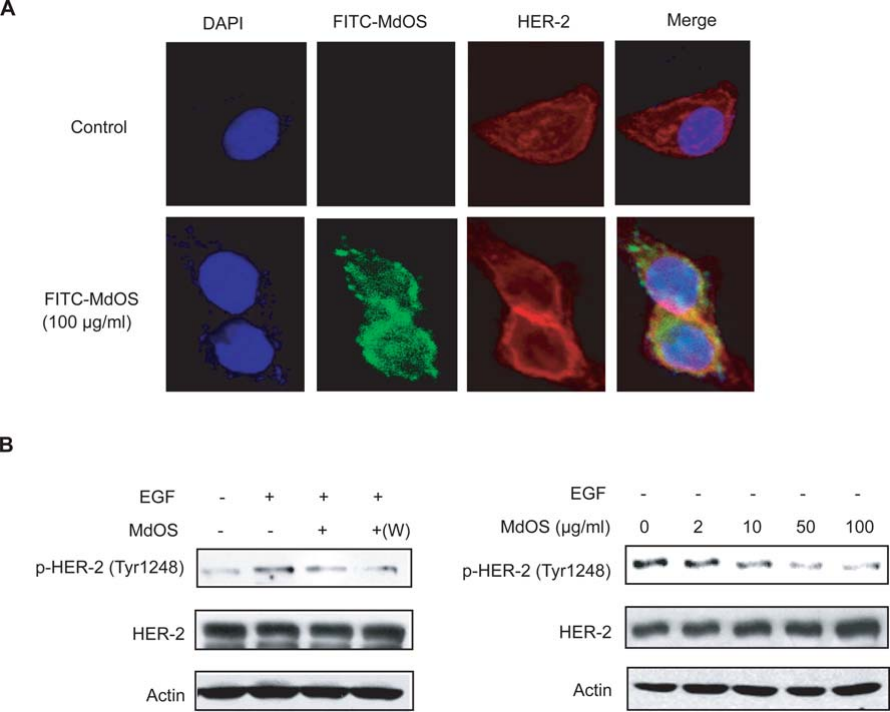
MdOS directly targets intracellular tyrosine kinase. A, Cell entry and location of MdOS. SK-OV-3 cells were treated as described in the materials and methods, and photographed by Leica TCS confocal microscope. Bar, 10 µm. B, *Left*, starved SK-OV-3 cells were incubated with 100 µg/ml MdOS for 6 h, followed by stimulated with EGF directly or washed three times with serum-free medium then stimulated with EGF (+w). *Right*, SK-OV-3 cells were plated in six-well plates. 24 h after plating, medium was replaced with serum-free medium supplemented with indicated concentrations of MdOS for 6 h.

Consensus view holds that oligosaccharides act extracellularly by inhibiting the interactions of growth factors to their receptors. In the aforementioned study, MdOS was interestingly and distinctly identified to arrest the kinase activity of growth factor receptors mainly via its intracellular action. To further confirm the MdOS-driven intracellular events, we first examined the cell entry of MdOS by using a FITC-labeled MdOS (FITC-MdOS, green) probe in SK-OV-3 cells[20]. As shown in Figure 3A, high levels of green fluorescence were detected in the MdOS-treated SK-OV-3 cells, while no visible green fluorescence was noted in untreated cells. Green fluorescence reached saturated upon 30-min exposure (data not shown), indicative of a rapid uptake of MdOS into cells. The fluorescence co-localization analysis further revealed that MdOS partially superimposed with HER-2 tyrosine kinase in SK-OV-3 cells, favoring appreciable entry of MdOS into cells.

To provide more direct evidence and rule out contributions of extracellular activity, we then carried out another subsequent experiments, by withdrawing the added MdOS prior to the exposure of growth factors and by depriving the growth factors from the experimental settings as well. As expected, MdOS inhibited the phosphorylation of HER-2 in SK-OV-3 cells in the MdOS-withdrawal sample similar to that in MdOS-unwithdrawal sample (Fig. 3B, left). Likewise, MdOS also blocked phosphorylated HER-2 in SK-OV-3 cells in growth factor-deprived settings (Fig 3B, right).

Together with our previous data in Figure 2, our results substantiate that MdOS directly inhibit cellular tyrosine kinase activity independent of extracellular activity, and strongly support that MdOS behaves distinctly from the conventional oligosaccharides.

### MdOS potently binds to the kinase domain of VEGFR2, HER-2 and EGFR

**Figure 4:**
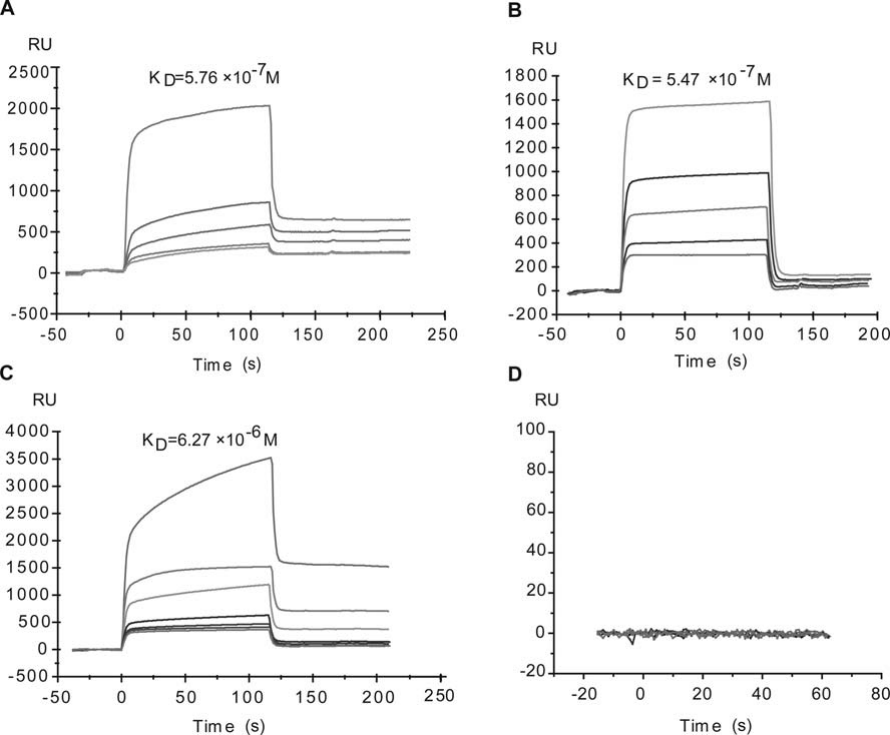
Interaction of VEGFR-2 (A) or HER-2 (B) or EGFR (C) or 6×his-tag (D) with MdOS by surface plasmon resonance analysis. VEGFR2 was injected at the concentrations of 2.56, 0.64, 0.32, 0.16 and 0.08 µM (from top to bottom); HER-2 was injected at the concentrations of 0.53, 0.26, 0.13, 0.07 and 0.03 µM (from top to bottom); EGFR was injected at the concentrations of 6, 3, 1.5, 0.75, 0.38, 0.19 and 0.09 µM (from top to bottom); 6×his-tag was injected at the concentrations of 100, 10, 1, 0.1, 0.01 µM. Sensorgram responses at equilibrium were plotted against each concentration of compounds and the equilibrium dissociation constant (K_D_) of the binding system was calculated using BIAeval software 3.1.

We next determined whether the inhibitory effect of MdOS on the activity of VEGFR2, HER-2 and EGFR is associated with the direct engagement with tyrosine kinases. SPR assay was employed to assess the interaction between MdOS and VEGFR2 or HER-2 or EGFR. For this, MdOS was immobilized on CM-5 sensor chips. The response of binding units of MdOS to VEGFR2 or HER-2 or EGFR was recorded. MdOS was noted to display a high-binding affinity for VEGFR2, yielding the equilibrium dissociation constant (K_D_) of 5.76×10^−7^ M (Fig. 4A). Similar results were observed in MdOS-HER-2 (Fig. 4B) and MdOS-EGFR binding settings (Fig. 4C), with K_D_ values of 5.47×10^−7^ M and 6.27×10^−6^ M, respectively.

To address whether the histine tagged on the proteins interacts with MdOS or not, binding between MdOS and 6×his-tag was examined. As shown in Figure 4D, no obvious association was found between MdOS and pure 6×his-tag. These results demonstrated that the His-tag moiety does not affect the binding ability of our protein to MdOS, permitting the identity of His-tagged proteins to the native ones in terms of the biological outcomes of their interactions with MdOS.

### MdOS functions as an ATP competitive inhibitor of VEGFR2, HER-2 and EGFR

**Figure 5:**
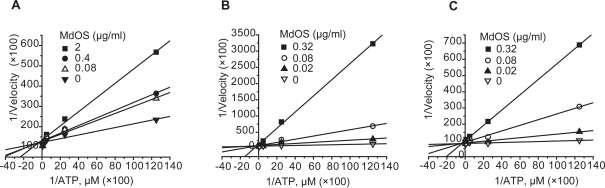
Lineweaver Burke plot of ATP competition. VEGFR2 (A) and HER-2 (B) and EGFR (C) kinase assays were performed as described in Materials and Methods in the presence of varying concentrations of ATP. Initial reaction velocity was expressed as the phosphorylation of poly(Glu, Tyr)_4∶1_ substrate. All x, y data sets were multiplied by 100 for purposes of graphical presentation.

Most small molecular inhibitors are designed to block kinase activity via binding to the ATP pocket of the enzyme. To examine whether MdOS also acts as an ATP competitive inhibitor, we evaluated the inhibitory potency of MdOS on kinase enzymatic activities using competitive inhibitory assay by introduction of different concentrations of ATP. As shown in Figure 5, Lineweaver-Burke plots for inhibition of VEGFR2, HER-2 and EGFR tyrosine kinases by MdOS with respect to ATP concentration showed all curves intersecting the y-intercept at zero, which is indicative of a competitive mechanism of inhibition.

### MdOS binds to the entrance of ATP binding pocket of VEGFR2 and EGFR

**Figure 6:**
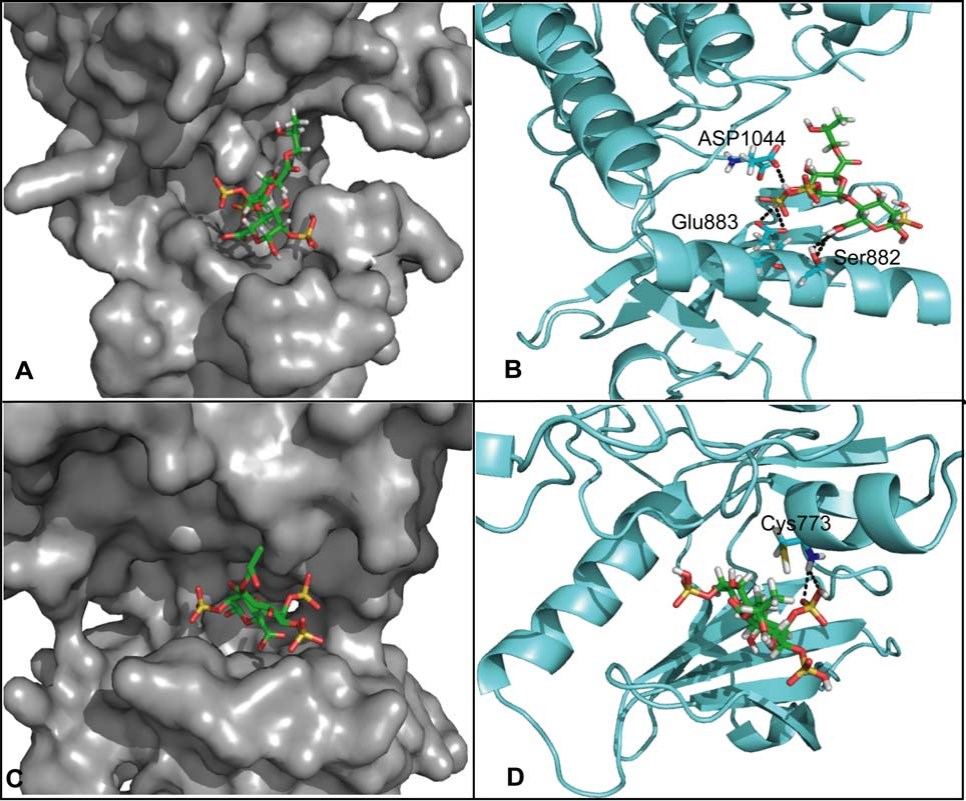
Predictive interaction between MdOS and VEGFR2 or EGFR by molecular docking. A, binding mode of MdOS and VEGFR2 kinase (PDB code: 1YWN). B, the detailed binding interactions between MdOS and VEGFR2 kinase. Hydrogen bonds are indicted by dashed lines. C, binding mode of MdOS and EGFR kinase (PDB code: 1XKK). D, the detailed binding interactions between MdOS and EGFR kinase. Hydrogen bonds are indicted by dashed lines.

In order to go further insights into interaction mode of MdOS with these kinase proteins at the atomic level, the automated docking simulation exemplified by MdOS-VEGFR2 and MdOS-EGFR interaction were performed. The crystal structures of human VEGFR2 and EGFR kinases were utilized as the target structures, with the disaccharide of MdOS as docking probe. In the model of VEGFR2, it was remarkably noted that different from the classic small molecule inhibitors that probed deeply into the ATP binding pocket, MdOS was docked at the entrance of the ATP-binding pocket of VEGFR2 (Fig. 6A). In a zoomed-in view, two sulfate moieties of MdOS are hydrogen-bonded to the sidechain carbonyl oxygen of Glu883 and Asp1044 respectively, and the carboxyl group of MdOS generates two hydrogen bonds with the hydroxyl group of Ser882 (Fig. 6B). In addition, the sugar moiety forms hydrophobic interactions with residues Phe843 and Glu883.

Similar binding mode was observed in MdOS and EGFR kinase. Likewise, MdOS also encompassed the entrance of the ATP pocket of EGFR (Fig. 6C), with one sulfate moiety hydrogen bonding to Cys773 (Fig. 6D). And the sugar moiety forms hydrophobic interactions with residues Asp776. All these allow MdOS to mask entrance of ATP-binding pocket, thereby blocking the engagement of ATP with VEGFR2 and EGFR, which further help understand the ATP-competitive inhibitory competence of MdOS.

### MdOS exhibits anti-angiogenic activities both in vitro and in vivo

VEGF and VEGFR2 signaling in endothelial cells is primarily responsible for tumor angiogenesis. MdOS preferably inhibited the proliferation of HMECs (data not shown) and particularly suppressed VEGFR2 phosphylation and subsequent downstream signaling in HMECs (Fig. 2E). In addition, MdOS also targeted HER-2, EGFR, FGFR and PDGFR that are directly or indirectly involved in tumor angiogenesis[21], [22], [23]. All these prompted us to hypothesize that MdOS might exhibit anti-angiogenic effects.

To address this issue, we first defined the appropriate concentrations and duration that is irrelevant to the cytotoxic effect of MdOS. For this, both MTT and BrdU incorporation assays were selected. MdOS demonstrated little inhibitory impact on cell viability within 12 h, even at concentration of 100 µg/ml (data not shown). Accordingly, the concentrations no more than 100 µg/ml and exposure time within 12- h were selected for the subsequent experiments.

#### MdOS arrests growth factors-induced proliferation in HMECs

**Figure 7:**
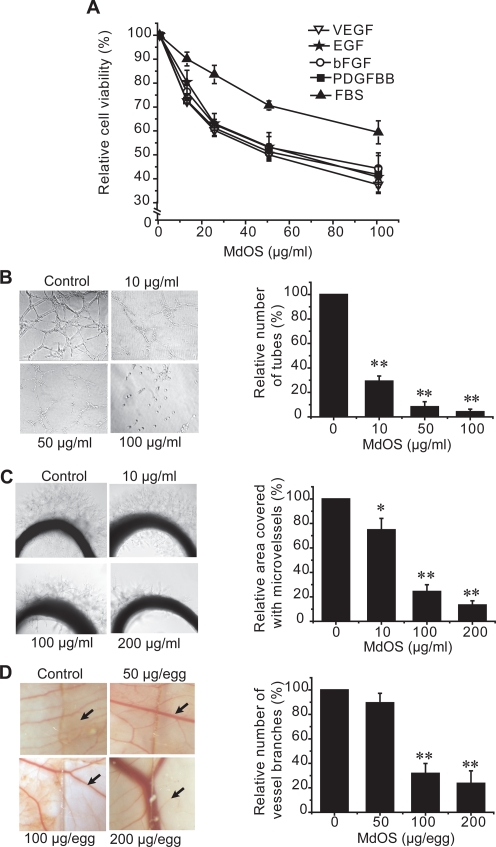
The inhibitory action of MdOS on angiogenesis. A, effect of MdOS on growth factor-stimulated proliferation of human microvascular endothelial cells (HMECs). HMECs proliferation was assayed as described in Materials and Methods. B, effect of MdOS against HMEC tube formation on Matrigel. HMECs were plated on Matrigel in medium with various concentrations of MdOS for 8 h. *Left*, representative photographs (100×) of three independent experiments. *Right*, quantification of the inhibitory activity of MdOS on tube formation. C, effect of MdOS on microvessel outgrowth arising from rat aortic ring. Aortic rings were embedded in Matrigel in 96-well plates, then fed with medium containing various concentrations of MdOS for 6 days. *Left*, representative photographs (100×) of three independent experiments. *Right*, the area of microvessels was quantified and normalized to untreated controls. D, effect of MdOS on angiogenesis in a chorioallantoic membrane model. Glasscover-slip saturated with MdOS or normal saline was placed areas between preexisting vessels in the fertilized chicken eggs and incubated for 48 h. *Left*, representative photographs (100×) of three independent experiments. The Glasscover-slip was placed on the right side of the field. *Right*, the number of vessel branches was quantified and normalized to untreated controls. Data shown are mean±SD from three independent experiments.*, P<0.05; **, P<0.01, versus control.

VEGF, EGF, bFGF and PDGF have been clearly identified as positive mediators of endothelial cell proliferation and angiogenesis[24]. We examined the effects of MdOS on growth factors-dependent HMEC cell proliferation. As expected, MdOS displayed comparable inhibitory activities against VEGF-, EGF-, bFGF- and PDGF-driven HMECs proliferation (IC_50_ values of 48.3 µg/ml, 61.0 µg/ml, 56.2 µg/ml and 62.7 µg/ml, respectively), whereas with less potency against FBS-mediated events (IC_50_ value >200 µg/ml) (Fig. 7A). These findings further substantiate the multi-targeted profiles of MdOS in HMECs and the likely potential of its anti-angiogenic efficacy.

#### MdOS disrupts HMEC tube formation on matrigel

As tube formation represents one of the late stage of angiogenesis, we next evaluated the effects of MdOS on tube formation of HMECs on a Matrigel substratum[25]. In the control group, HMECs formed a mesh of tubes within 8 h. The tube structures were severely and concentration-dependently disrupted when cells were exposed to MdOS. Notably, HMECs treated with lower concentrations (10 and 50 µg/ml) of MdOS differentiated into short tubes but were unable to form meshes, whereas those treated with higher concentration of MdOS (100 µg/ml) remained dotted on the Matrigel (Fig. 7B).

#### MdOS inhibits microvessel outgrowth from rat aortic rings

We further evaluated the anti-angiogenic effects of MdOS in an ex vivo aorta sprout outgrowth assay [25]. We found that new microvessels began to grow when control cultures were incubated for 6 days. Treatment with MdOS (50, 100 and 200 µg/ml) resulted in a notable suppression of microvessel formation versus the control (Fig. 7C).

#### MdOS abrogates neovascularization in a CAM model

To determine whether anti-angiogenic effects of MdOS could be recapitulated *in vivo*, we extended our studies to a CAM model [25]. As shown in Figure 7D, the normal branching pattern of blood vessels formed over a 2 day incubation in the control group, whereas MdOS at 200 µg/egg completely blocked this angiogenesis.

## Discussion

The high frequency of genetic alterations in cancer cells resulting in permanent activation of protein tyrosine kinases has led to the positioning of these kinases as therapeutic targets[26]. It is believed that there is multi-level cross-stimulation among targets along several pathways of signal transduction that lead to malignancy in tumor. Blockage on one pathway, as most single-target agents do, other pathways are compensatorily allowed to act as salvage or escape mechanisms [27]. As such, a multi-targeted approach where several pathways are selectively and simultaneously suppressed at the RTK level represents an attractive therapeutic strategy. Moreover, arrest of multiple signal pathways favors the overcome of resistance that commonly occurs in single-targeting monotherapy[28]. Therefore, the multi-targeted approaches are anticipated through either combination of selectively defined agents or by a single agent with multiple targets. The latter, in this scenorio, offers the favorable advantages over the combined therapy due to the multi-functional convenience, easier dosing regimens, and potential lower costs.

With an increasing understanding of SAR-based efficacy *versus* toxicity of multi-targeted PTK inhibitors, these benefits emerge to weight against the potential limitation, for instance, the mutations in residues of ATP-binding cleft disable the active access of the established inhibitors to the ATP-binding domain, thereby offering the inevitable resistance [29], [30]. A logical approach, therefore, would involve compounds that bind to residues beyond or far off the ATP-binding cleft. Of note, the tyrosine kinase inhibitors are more often the kind of small molecules that have been directed towards this ATP-binding pocket. The therapeutic options are considered with those agents of structurally novel skeletons.

MdOS is structurally defined as a 1,4-linked β-D-mannuronate of pyranohexuronic acid residues with the reducing end oxidized to mannurarate, bearing an average of 1.5 sulfates per sugar residue at the 2-hydroxyl, partial 3-hydroxyl with partial esterification at 6-carboxyl groups. This structure offers MdOS appreciable negative charges, which allows MdOS to afford a high-affinity interaction with PTK in a broad spectrum manner. Our ELISA-based kinase inhibitory outcomes that MdOS broadly inhibited PTKs, together with our cellular assays that MdOS inhibited PTKs phosphorylation and downstream signaling, substantiate this story. The concomitant *in vivo* anti-angiogenic efficacies of MdOS should be the synergistic outcomes of this broad-spectrum inhibitory action. Notably, the unique binding mode by the simulation exercise that MdOS hydrogen bonded to the residues of the entrance rather than those of ATP-binding pocket helps, from a theoretical view, to understand the likely distinct pharmacological profiles from small molecules in general and, the potential value against small molecule-evoked resistance in particular. Of course, much hope lies on further investigation.

Although electrostatic charges are viewed to initiate attachments between sugars and proteins, the structure-based conformation likely dominates the functions. The alternative findings that some other marine-derived oligosaccharides, even bearing the same negative charges but with different sugar compositions, did not generate the likelihood of functions as MdOS did (data not shown), help reinforce this view. The structural components responsible for interactions between PTK and sulfated oligosaccharides have not been well characterized, but the nature of the sugar scaffold seems to be critical, and distribution of sulfation seems essential.

In summary, this is the first time to disclose that MdOS, a marine-derived oligosaccharide, functions as a broad spectrum of tyrosine kinase inhibitor. It is canonically viewed that oligosaccharides act extracellularly by inhibiting the interactions of growth factors to their receptors [31]. However, distinctly from this commonly accepted concept, MdOS represents an alternate mechanism via arresting intracellularly the kinase activity of growth factor receptors. As yet, it cannot exclude the partial extracellular activity of MdOS on angiogenesis, but we focus on the intracellular function of MdOS in this study. The appreciable pharmacological profiles, the distinct action of mode as well as the low cytotoxic settings (data not shown) underscore the importance of MdOS as a new lead scaffold in anti-cancer drug development. With research continuing to dig the insights into the structure-based machinery of this kind of oligosaccharide, it is hoped that the future will add impetus to the search for carbohydrate-based multi-targeted PTK inhibitors in cancer therapy.

## Materials and Methods

### Compound

The oligomannuronic acid was degraded by hydrogen peroxide from polymannuronate blocks obtained from sodium alginate. The MdOS compound was further obtained by semi-synthesis following sulfate modification by reacting its precursor with ClSO_3_H in formamide after partially esterified at the 6-carboxyl groups. Briefly, oligomannuronate was mixed with propylene oxide at a certain ration, and the reaction was catalyzed by NaOH at 45°C for 3 h. Then the esterified products were added to sulfating reagents containing formamide and ClSO_3_H, and reacted for 3 h. The pH of the products was adjusted to 7.0 with NaOH, and desalted using Sephadex G-10. The product peak was pooled and freeze-dried. The molecular weights of MdOS and its precursor were analyzed by High Performance Gel Permeation Chromatography (HPGPC) using a TSK G3000PWxl column (300 mm×7.8 mm) (Tosoh, Tokyo, Japan). Stock solutions were prepared in saline solution at 10 mg/ml and then diluted to the desired concentration with saline solution.

### Cells and cell culture

Human squamous cancer cells A431, human ovarian cancer cells SK-OV-3, human micro-vascular endothelial cells (HMECs) and mouse fibroblast cells NIH-3T3 were obtained from the American Type Culture Collection (Rockville, MD). NIH-3T3/neu and NIH-3T3/flk-1 were established as described previously[32]. SK-OV-3 and NIH-3T3 cells were maintained in high-glucose DMEM (Gibco, Grand Island, NY) with 10% FBS, 100 IU/ml benzylpenicillin, and 100 mg/L streptomycin. A431 cells were maintained in DMEM with 10% FBS, 4 mM L-glutamine, 100 IU/ml benzylpenicillin, and 100 mg/L streptomycin. HMECs were grown in MCDB-131 medium containing 10% FBS. NIH-3T3/neu and NIH-3T3/flk-1 cells were maintained in DMEM (Gibco, Grand Island, NY) with 10% FBS, 100 IU/ml benzylpenicillin, 100 mg/L streptomycin and 600 mg/L G418. All cells were cultured in humidified atmosphere of 95% air and 5% CO_2_ at 37°C.

### Animals

Sprague-Dawley rats (6-week-old) were provided by Shanghai Laboratory Animal Center, Chinese Academy of Sciences. The use of animals was approved by the Institute animal reviews boards of Shanghai Institute of Materia Medica, Chinese Academy of Sciences, with confirm adherence to the ethical guidelines for the care and use of animals.

### ELISA kinase assay

The kinase domain of EGFR, HER-2, VEGFR2, c-Kit, FGFR1, FGFR2 and c-Src were expressed using the Bac-to-Bac™ baculovirus expression system (Invitrogen, Carlsbad, CA, USA) and purified on Ni-NTA columns (QIAGEN Inc., Valencia, CA, USA) as previously described[33]. Recombinant PDGFRβ and VEGFR1 proteins were obtained from Upstate Biotechnology (Lake Placid, NY. #14-463 and #14-562, respectively). The tyrosine kinase activities of the purified EGFR, HER-2, Flt-1, VEGFR2, PDGFRβ, c-Kit, FGFR1, FGFR2 and c-Src kinase domains were determined using ELISA methodology described previously[34]. Briefly, 50 µl 10 µM ATP solution diluted in kinase reaction buffer (50 mM HEPES pH 7.4, 20 mM MgCl_2_, 0.1 mM MnCl_2_, 0.2 mM Na_3_VO_4_, 1 mM DTT) was added to each well. Various concentrations of MdOS diluted in 10 µl buffered saline were added to each reaction well. 10 µl buffered saline was used as the vehicle control. The kinase reaction was performed in triplicate and initiated by adding purified tyrosine kinase proteins diluted in 40 µl of kinase reaction buffer. After incubation for 60 min at 37°C, the plate was washed three times with Phosphate Buffered Saline (PBS) containing 0.1% Tween 20 (T-PBS). Next, 100 µl anti-phosphotyrosine (PY99) antibody (1∶500, Santa Cruz Biotechnology, Santa Cruz, CA) diluted in T-PBS containing 5 mg/ml BSA was added and the plate was placed at at 37°C for 30 min. After the plate was washed three times, 100 µl Horseradish peroxidase-conjugated goat anti-mouse IgG (1∶2000, Calbiochem, San Diego, CA) diluted in T-PBS containing 5 mg/ml BSA was added and the plate was reincubated at 37°C for 30 min. The plate was washed, then 100 µl citrate buffer (0.1 M, pH 5.5) containing 0.03% H_2_O_2_ and 2 mg/ml o-phenylenediamine was added and samples were incubated at room temperature until color emerged. The reaction was terminated by adding 50 µl of 2 M H_2_SO_4_, and the plate was read using a multiwell spectrophotometer (VERSAmax™, Molecular Devices, Sunnyvale, CA,USA) at 492 nm. The inhibition rate (%) was calculated with the formula: [1-(A492/A492 control)]×100%. IC_50_ values were calculated from the inhibitory curves. For ATP competitive assay, conditions were the same as above except that varying concentrations of ATP were added in the absence or presence of a single concentration of MdOS to generate ATP concentration curves.

### Western blot analysis

Cells were grown to half confluence in six-well plates, starved in serum-free medium for 24 h, and then exposed to MdOS at concentrations of 2, 10, 50 and 100 µg/ml for 6 h. For analysis of receptor tyrosine kinase phosphorylation and downstream signal transduction pathways, cells were stimulated with 50 ng/ml EGF or VEGF (R&D Systems, Minneapolis, MN, USA) for 15 min at 37°C at the end of MdOS treatment. Western blot analyses were subsequently performed as previous described[33], with the antibodies against phospho-EGFR (Tyr1068) (#2234), phospho-HER-2 (Tyr1248) (#2247), phospho-VEGFR2 (Tyr996) (#2474), VEGFR2 (#2472), phospho-ERK1/2 (#9101), ERK1/2 (#9102), phosphor-AKT (Ser473) (#9271) and AKT (#9272) (Cell Signaling Technology, Beverly, MA, USA), EGFR (sc-03), HER-2(sc-284) and actin (sc-8432) (Santa Cruz Biotechnology, Santa Cruz, CA).

### Preparation of the fluorescence-tagged MdOS

MdOS was labeled with fluorescein isothiocyanate (FITC) as previously described[20], [35] (Chemical Journal of Chinese Universities 2002; 23(9):1704–08). Briefly, 400 mg MdOS was dissolved in PBS (pH 8.0) followed by mixing with 400 mg tyramine and 150 mg NaH3BCN. The mixture was stired at 37°C for 96 h. The solution was then loaded on a Sephadex G25 column to isolate MdOS-Tyr. Then 200 mg MdOS-Tyr and 25 mg FITC (Sigma-Aldrich) were dissolved in 0.5 M NaHCO3 (pH 8.5), and reacted at room temperature overnight. The solution was then loaded on a Sephadex G25 column to isolate FITC-labeled MdOS (FITC-MdOS) from unreacted FITC. The structure of the compound synthesized was confirmed by UV-VIS spectra and 1H-NMR.

### Immunofluorescence assay

For fluorescence staining, SK-OV-3 cells were plated on glass culture slides at a density of 1×10^5^ cells/ml in DMEM medium containing 10% FCS. The medium was replaced with serum-free DMEM containing 100 µg/ml FITC-MdOS (green) or normal saline for 6 h. Fixed preparations were obtained by exposing cells on culture slides to 4% paraformaldehyde in PBS for 10 min at 25°C, followed by washing three times with PBS containing 0.2% Triton X-100 for 5 min. For HER-2 proteins staining, cells were blocked with 5% bovine serum albumin for 30 min, then incubated with HER-2 antibody in 3% BSA (1∶200) for 30 min, and followed by coincubation with Alexa 633-conjugated secondary antibody diluted in 3% BSA (1∶ 500) for 30 min. Then 1 µg/ml DAPI was added for visualization of nuclear acid. coverslips were slightly dried for a few minutes and mounted on the slides with anti-fade agent, and pictures were obtained using Leica TCS confocal microscope (Leica, Deerfield, IL, USA).

### Surface Plasmon Resonance Analysis

The kinetics and specificity of the binding reactions between MdOS and VEGFR2 or HER-2 or EGFR or 6×his-tag were carried out with the BIAcore×surface plasmon resonance apparatus. Briefly, MdOS was immobilized on CM5 sensor chips according to the Ligand Thiol protocol described in the application Handbook. To assess real-time binding capacity, 35 µl of soluble VEGFR2 or HER-2 or EGFR or 6×his-tag was injected over the sensor chip surface with the immobilized MdOS, followed by washed with HBS-EP buffer for 5 min. The sensor chip surface was regenerated using 60 µl NaCl (2 M). All binding experiments were performed at 25°C with a constant flow rate of 10 µl/min HBS-EP. For binding affinity assessment, the association phase was allowed to proceed to equilibrium. To correct for nonspecific binding and bulk refractive index change, a blank channel (FC2) without oligosaccharide was employed as a control for each experiment. Sensorgrams for all binding interactions were recorded in real time and analyzed after subtracting that from the blank channel. Changes in mass due to the binding response were recorded as resonance units (Ru). Binding kinetics and affinities were determined by SPR using BIACORE software 3.1.

### Molecular modeling

The crystal structure of VEGFR2 kinase in complex with a known inhibitor was retrieved from the Brookhaven Protein Data Bank (PDB entry 1YWN). The crystal structure of EGFR kinase in complex with lapatinib was retrieved from the Brookhaven Protein Data Bank (PDB entry 1XKK). The molecular geometry of MdOS disaccharide was modeled using the ISIS draw 2.5 program (Available from http://www.mdl.com). The initial structures were minimized using molecular mechanics with the Tripos force field [36] with Gasteiger–Marsilli charges[37], and the energy convergence gradient value was set to 0.005 kcal/(mol Å). Docking studies using the DOCK 4.0 program (Kuntz group, San Francisco, CA, USA) [38]were performed to predict the binding of MdOS to VEGFR2 or EGFR. Kollman-all-atom charges and Geisterger-Hückel charges were assigned to proteins and the MdOS, respectively. Spheres were generated by the AutoMS and sphgen programs encoded in DOCK4.0 to describe the shape of the binding site. The hydrophobic and electrostatic properties of the binding site were calculated by the program Grid4. And the conformational flexibility of MdOS was considered during the docking calculation. Finally, the evaluation with the lowest binding energy was used to analyze ligand pose and the interaction model of MdOS/VEGFR2 or MdOS/EGFR was produced using the LIGPLOT program based on the docked complex structure[39].

### Growth factor-induced cell proliferation assays

HMEC Cells were seeded in 96-well tissue culture plates. On the next day, cells were incubated in serum-free medium with 1% FBS for 24 h. Cells were then exposed to various concentrations of MdOS and growth factors (50 ng/ml VEGF, EGF, bFGF, PDGFBB or 10% FBS) were added. Cells were further cultured for 2 days. Cell proliferation was determined using sulforhodamine B (SRB; Sigma, St. Louis, MO, USA) assay.

### Tube formation assay

96-well plates were coated with 60 µl liquid Matrigel (Eecton Dickinson, Bedford, MA, USA) per well and incubated at 37°C for 1 h to promote gelling. HMECs (1.5×10^4^/well) were seeded on the Matrigel and cultured in MCDB-131 with various concentrations of MdOS for 8 h at 37°C and 5% CO_2_. Images were recorded by an inverted phase contrast microscope (Olypus, IX70, Japan), the enclosed networks of complete tubes from five randomly chosen fields were counted and photographed.

### Rat Aortic Ring Assay

The aortas were harvested from 6-week-old Sprague-Dawley rats. Each aorta was cut into 1-mm slices and embedded in 70 µl Matrigel in 96-well plates. The aortic rings were then fed with 100 µl of M199 with different concentrations of MdOS or vehicle alone, and photographed on day 6. The quantity of microvessels is valued by relative area covered with microvessels using Image-Pro Express.

### Chicken Chorioallantoic Membrane Assay

Groups of 10 fertilized chicken eggs were incubated in a humidified egg incubator (Lyon, Chula Vista, CA) maintained at 37°C and 50% humidity and allowed to grow for 7 days. Gentle suction was applied at the hole located at the broad end of the egg to create a false air sac directly over the chicken chorioallantoic membrane, and a 1-cm^2^ window was removed from the egg shell immediately. Glass coverslips (0.5×0.5 cm) saturated with compounds or normal saline was placed on areas between preexisting vessels and the embryos were further incubated for 48 h. The neovascular zones beside the glass coverslips were photographed under a stereomicroscope (Leica, MS5, Heerbrugg, Switzerland) and printed out as 5×7 in^2^ prints. Vessel branches <3 mm long in five random 1×1 in^2^ zones per pictures were counted and five eggs were chosen from each group.

### Statistical analysis

All data are represented as mean±SD. The significance of differences was assessed by Student's t test. P-values of <0.05 were regarded as statistically significant.
